# Exploration of Dynamic Elastic Modulus Changes on Glioblastoma Cell Populations with Aberrant EGFR Expression as a Potential Therapeutic Intervention Using a Tunable Hyaluronic Acid Hydrogel Platform

**DOI:** 10.3390/gels3030028

**Published:** 2017-07-13

**Authors:** Hemamylammal Sivakumar, Roy Strowd, Aleksander Skardal

**Affiliations:** 1 Wake Forest Institute for Regenerative Medicine, Wake Forest School of Medicine, Medical Center Boulevard, Winston-Salem, NC 27157, USA; hsivakum@wakehealth.edu; 2 Department of Neurology, Wake Forest School of Medicine, Medical Center Boulevard, Winston-Salem, NC 27157, USA; rstrowd@wakehealth.edu; 3 Virginia Tech-Wake Forest School of Biomedical Engineering and Sciences, Wake Forest School of Medicine, Medical Center Boulevard, Winston-Salem, NC 27157, USA; 4 Department of Cancer Biology, Wake Forest School of Medicine, Medical Center Boulevard, Winston-Salem, NC 27157, USA; 5 Comprehensive Cancer Center at Wake Forest Baptist, Wake Forest Baptist Health Sciences, Medical Center Boulevard, Winston-Salem, NC 27157, USA

**Keywords:** glioblastoma, elastic modulus, tumor growth, epidermal growth factor receptor, hydrogel

## Abstract

Glioblastoma (GBM) is one of most aggressive forms of brain cancer, with a median survival time of 14.6 months following diagnosis. This low survival rate could in part be attributed to the lack of model systems of this type of cancer that faithfully recapitulate the tumor architecture and microenvironment seen in vivo in humans. Therapeutic studies would provide results that could be translated to the clinic efficiently. Here, we assess the role of the tumor microenvironment physical parameters on the tumor, and its potential use as a biomarker using a hyaluronic acid hydrogel system capable of elastic modulus tuning and dynamic elastic moduli changes. Experiments were conducted to assess the sensitivity of glioblastoma cell populations with different mutations to varying elastic moduli. Cells with aberrant epithelial growth factor receptor (EGFR) expression have a predilection for a stiffer environment, sensing these parameters through focal adhesion kinase (FAK). Importantly, the inhibition of FAK or EGFR generally resulted in reversed elastic modulus preference. Lastly, we explore the concept of therapeutically targeting the elastic modulus and dynamically reducing it via chemical or enzymatic degradation, both showing the capability to reduce or stunt proliferation rates of these GBM populations.

## 1. Introduction

Glioblastomas are malignant tumors that arise from astrocytes, the star-shaped cells that make up the glue-like or supportive tissue of the brain [1]. Glioblastoma (GBM) is the most common malignant tumor of the central nervous system, and it occurs in every 3.19 per 100,000 people [1,2]. The survival rate is very low, as less than 5% of the patients live beyond five years from the time of diagnosis [2]. The disease is challenging to treat, as eliminating the tumor completely through surgery is near to impossible, and minimal chemotherapeutics reach the tumor due to their inability to penetrate the tight junctions of the blood–brain barrier. Moreover, glioblastomas universally reoccur, even after maximally aggressive treatment. These challenges are the primary reasons for such poor survival rates in glioblastoma patients [3].

GBM tumors are comprised of heterogeneous subpopulations of genetically and perhaps microenvironmentally distinct regions, both of which may be causes for why some GBM cells evade treatment and drive recurrence [1,3]. The difference between the brain microenvironment and other tissues might explain the commonly observed scenario of glioblastoma rarely metastasizing outside the brain, but rather preferring to aggressively invade within the confines of the brain [4]. The extracellular matrix (ECM) in the brain is substantially different compared to other tissues in the body. Major matrix components, including fibrous matrix proteins like collagen and fibronectin, which are present in abundance in other organs, are completely absent or minimally present in the brain. Instead, hyaluronic acid (HA) is the major component in the brain ECM [4,5]. This unique composition of HA plays an important role in forming the GBM tumor microenvironment [4,6,7,8,9]. The brain ECM was once considered a simple supporting system that provided physical adhesion to cells, but is now understood to play a vital role in neurological development, function and degeneration. Mechanical properties differ between normal and diseased tissues, and this has been used for diagnostic purposes for many years [10]. Change in the rigidity of the brain ECM has been implicated in neuropathological diseases such as Alzheimer’s, where it has been shown that the brain tissue of Alzheimer’s patients is reduced in elastic modulus compared to healthy controls [11]. Research conducted in the past decade has demonstrated that physical ECM cues play an important role in contributing to the regulation of tumor cell proliferation and migration, including glioma cells [12]. As such, ECM rigidity provides a transformative and microenvironmental cue to the tumor cells in the brain [7]. Moreover, the tissue elastic modulus has been used in assessing the prognosis of other cancers, such as breast cancer and liver cancer, for decades [13,14].

The interaction of cells with ECM proteins generates intracellular signals that regulate growth, survival, and migration. The integrin receptors of cells function as the link between ECM proteins and the cellular actin cytoskeleton [15]. In this process, focal adhesion kinase (FAK) is the coordinator of extracellular inputs with intracellular signaling pathways [16]. FAK colocalizes with integrin receptors at cell–substratum contact sites in the cells, and the kinase signaling activity is controlled by cellular binding to ECM proteins, as well as the activation of other cell surface receptors [15,17]. Elevated levels of FAK expression and activation have been reported in GBM and have also been implicated in GBM cell proliferation and migration [16,17]. Additionally, GBM has often been linked to upregulated or aberrant epidermal growth factor receptor (EGFR) expression [18]. EGFR activation leads to autophosphorylation and signal transduction, which controls cell proliferation, gene transcription and apoptosis. Upregulation and mutation of EGFR is implicated in a variety of tumors with progression to invasion, and as such, EGFR and downstream signaling molecules are targets for therapeutic interventions. Of the various mutant EGFR forms seen in GBM, the most common is ΔEGFR (also named EGFRvIII), which leads to ligand-independent constitutive activation [19,20,21]. Some GBM tumors express a mutated form of EGFR, with a tandem duplication mutation resulting in a 190-kDa EGFR replacing a 170-kDa EGFR. This heavier EGFR is also constitutively active and highly oncogenic [21]. Importantly, cross talk between EGFR signaling and FAK signaling has been observed in cell migration and metastasis of cancers [22]. Targeting of these receptor mutations has been widely explored as a potential therapeutic intervention in cell and animal models, and recently in human clinical trials. Unfortunately, these efforts have been for the most part unsuccessful. Notably, there was a recent phase III study of what looked like a very promising EGFRvIII vaccine that failed [23]. This failure and others indicate that new approaches and alternative therapeutic targets may be necessary in order to make headway against this disease.

Glial cells do not typically migrate in the soft brain tissue, but the stiffening of the ECM can facilitate more migration and invasion [24,25]. As such, the physical parameters of the brain tumor microenvironment may serve as potential targets of intervention. Previous studies have described how tissue elasticity can influence determination of the lineage of a differentiating cell, thus affecting a wide range of cell function and morphology, such as the lineage specification of adult stem cells [26] and maintenance of the therapeutic biomarkers of adult stem cells [27]. Moreover, the tissue elastic modulus has been used as a biomarker in diseases such as prostate cancer and cardiovascular diseases [28,29]. There is a vast scientific literature stressing the importance of the tumor cell and the microenvironment interaction [30,31,32]. Indeed, in recent studies, GBM cells responded differently to the environment elastic modulus of polyacrylamide substrates in vitro, altering proliferation and invasion behaviors [33].

Here we describe the use of a tunable hyaluronic acid-based hydrogel system [34,35,36,37,38,39] to more closely resembling the brain’s ECM composition, and assess the effects of elastic modulus manipulation on a panel of GBM cell populations of aberrant EGFR expression profiles. In particular, we query how elastic modulus influences cell proliferation and FAK expression. After, we demonstrate that elastic modulus-sensitive proliferation rates can be reduced or reversed via inhibition of either FAK or EGFR signaling, suggesting coordination between these pathways in these GBM populations. Lastly, we explore the use of dynamically targeting the microenvironment elastic modulus as a potential therapy to reduce or stop GBM cell proliferation rates. We believe that the tumor microenvironment physical parameters, rather than the cells themselves, may offer a future novel target for the therapeutic intervention of GBM.

## 2. Results and Discussion

### 2.1. Elastic Modulus-Dependent Proliferation Assays

**Figure 1:**
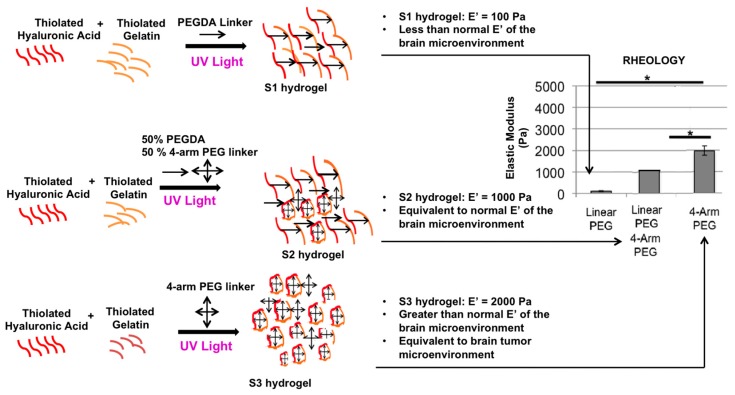
Tuning of hydrogel formulations by cross-linker manipulation. The three elastic moduli (100, 1000 and 2000 Pa) employed in S1, S2, and S3 hydrogels are obtained by modulating ratios of linear versus four-arm PEG-based cross-linkers. The elastic modulus corresponding to an environment less than normal brain elastic modulus (S1), normal brain elastic modulus (S2), and the brain tumor microenvironment (S3). Statistical significance: * *p* < 0.01 between hydrogel groups.

**Figure 2:**
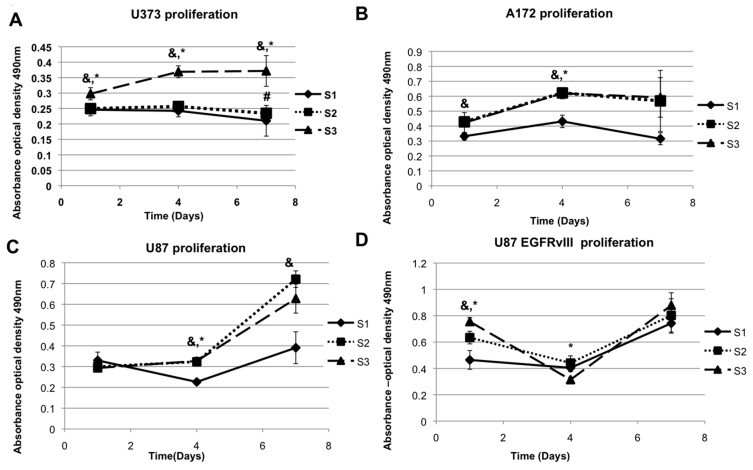
Cell proliferation rates as an effect of elastic moduli. U373 cells (**A**), A172 cells (**B**), U87 (**C**), and U87 EGFRvIII cells (**D**). * denotes *p* value is less than 0.05 when S1 and S3 values were compared. & denotes *p* value is less than 0.05 when values of S2 and S3 were compared and # denotes when *p* value is less than 0.05 when S1 and S2 values where compared.

There are several studies which have demonstrated that glioblastoma cell lines like U373 and U87 change proliferation and migration behaviors when cultured on a stiffer material [40]. These experiments have primarily been performed using polyacrylamide substrates or collagen-based gels, neither of which are representative of the HA-enriched brain tumor microenvironment. Hence, we employed a HA-based hydrogel system, with which we have previously created a variety of 3D tissue and tumor models [41,42,43,44,45] to perform elastic moduli-sensitive proliferation assays with four glioblastoma cell lines; U87-MG, U87-EGFRvIII (a U87 cell line genetically transfected to express the EGFRvIII mutation), U373, and A172 cells. We created hydrogels with three specific elastic moduli, ***E’***; 100 Pa (***E’*** S1, which is less than the elastic modulus of the normal brain), 1000 Pa (***E’*** S2, which is equivalent to the elastic modulus of the normal brain) and 2000 Pa (***E’*** S3, which is higher than normal brain tissue elastic modulus and equivalent to glioma tissue elastic modulus) [46,47] realms for this experiment by using a linear polyethylene glycol (PEG) cross-linker in S1, a four-arm PEG cross-linker in S3, or an equal combination of both in S2 (Figure 1). The experiment was done in a 2.5D model with cells being placed on S1, S2 and S3 hydrogels, after which cell proliferation rates were measured by MTS assays on day 1, 4 and 7 after seeding. It can be observed in Figure 2, that all the four cell types (U373, A172, U87, and U87 EGFRvIII) have a strong preference for hydrogels with higher elastic modulus (S3). The four cell lines mentioned above also share a common feature of aberrant EGFR expression: Upregulation (U373) or a mutation of EGFR (EGFRvIII in U87 EGFRvIII and 190-kDa mutation seen in A172). U87 is the outlier with a preference for a stiffer environment without EGFR upregulation or an EGFR mutation. In summary, the cell lines U87-MG, U373-MG, U87 EGFRvIII, and A172 all proliferated at a higher rate in stiffer hydrogels compared with softer hydrogels. As controls for each cell line, proliferation assays were also performed on tissue culture plastic (Figure S1). In each cell line, the proliferation of plastic was generally significantly increased compared to hydrogel conditions. Importantly, this result is not unexpected, as it is well documented that many cells proliferate more slowly on softer, more in vivo-like conditions [42,48]. The decreased proliferation rate on the HA hydrogels may be an indication of a cell culture system more like that of in vivo biology.

### 2.2. The Role of FAK in Elastic Modulus-Dependent Cell Proliferation

**Figure 3:**
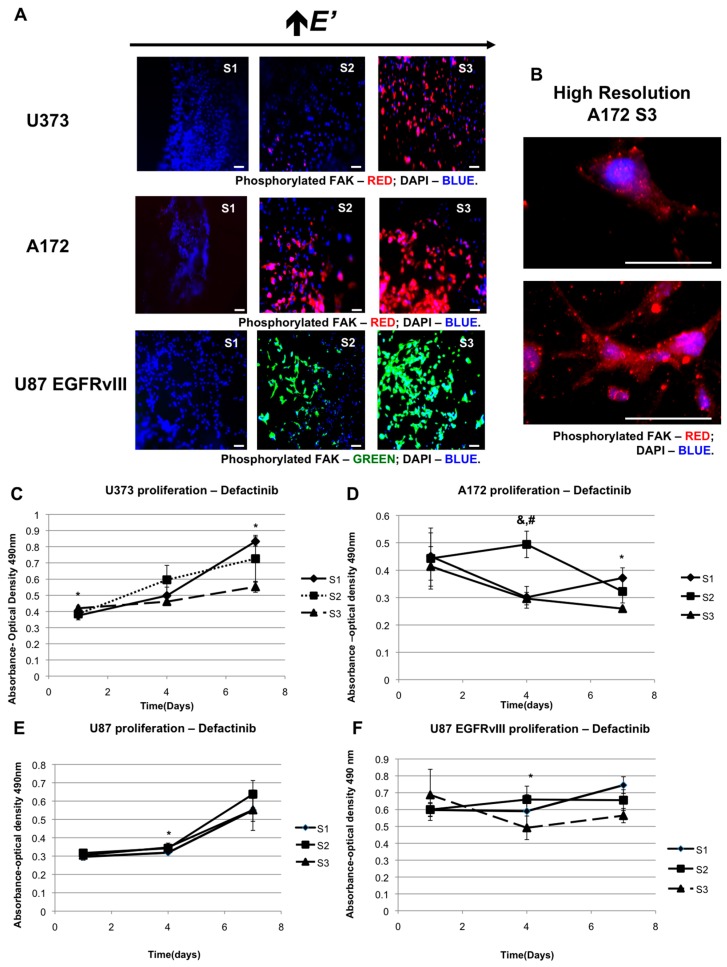
Focal adhesion kinase (FAK) expression increases with elastic modulus. (**A**) Immunofluorescent staining for phosphorylated FAK in U373 cells, A172 cells and U87EGFRvIII cells at S1, S2, and S3. Scale bars—100 μm; (**B**) Increased resolution imaging of FAK expression at S3. Scale bars—50 μm. (**C**–**F**) Proliferation of glioblastoma (GBM) cell types on varying elastic moduli under inhibition of FAK phosphorylation with defactinib: U373 cells (**C**), A172 cells (**D**), U87 (**E**) and U87 EGFRvIII cells (**F**). * denotes *p*-value was less than 0.05 when S1 and S3 values were compared, & denotes *p*-value was less than 0.05 when values of S2 and S3 were compared, and # denotes when the *p*-value was less than 0.05 when S1 and S2 values were compared.

Cells sense their surrounding through integrin-based adhesion complexes. These complexes are tightly linked with the cell’s actin cytoskeleton system, and comprise the cellular machinery that help the cells in recognizing the biochemical components of the extracellular matrix along with the physical characteristics of the ECM, such as its mechanical strength and its pliability [49]. FAK is one of the integral components of the adhesion complexes, and is a key intermediary in numerous integrin-originated signaling pathways [49]. Based on these observations, we hypothesized that the cells deduce and respond to their environment of higher elastic modulus through FAK phosphorylation. First, we queried whether FAK phosphorylation expression depended on the elastic modulus of the hydrogels through immunofluorescence studies. As can be seen in Figure 3A*,* FAK phosphorylation increased as the elastic modulus of the hydrogels increased. This suggests that FAK phosphorylation plays a vital role in the cells’ ability to sense the increasing elastic modulus of their environment. This led to our hypothesis that blocking FAK phosphorylation could influence the stiffness predilection of the cells. We tested this hypothesis by blocking FAK phosphorylation with defactinib (VS-6063, PF-04554878), an FAK phosphorylation inhibitor. Specifically, the drug inhibits the phosphorylation of FAK at Y397 position, which is upregulated in glioblastoma [50]. We repeated the elastic modulus-dependent proliferation assays with 100 nM defactinib. Figure 3C–F shows that when FAK phosphorylation is blocked, the three cell lines with aberrant EGFR expression, U373, A172 and U87 EGFRvIII, appear to have a reversal of ***E’*** preference, as evidenced by the higher proliferation of cells on hydrogels of lower ***E’*** S1. The U87 cells, which do not have EGFR upregulation or an EGFR mutation, have no such reversal.

### 2.3. The Effect of EGFR Inhibition on Elastic Modulus-Dependent Cell Proliferation

**Figure 4:**
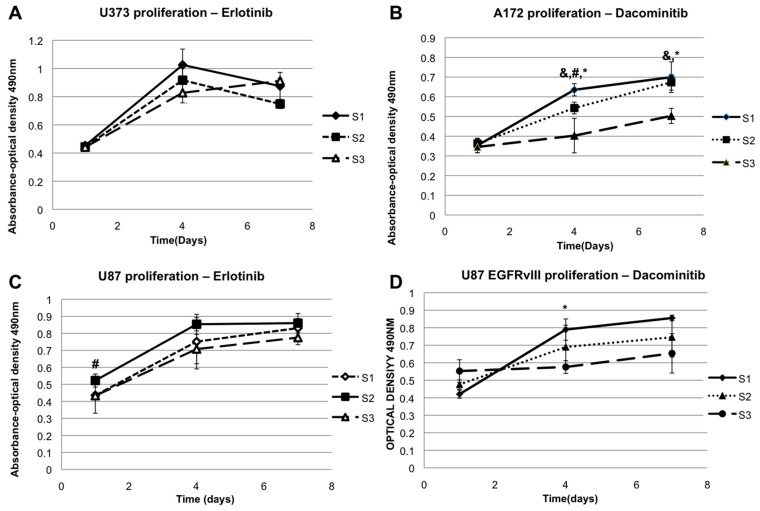
Proliferation of GBM cell types on varying elastic moduli under inhibition of epidermal growth factor receptor (EGFR) inhibitors: U373 cells with erlotinib (**A**), A172 cells with dacomitinib (**B**), U87 with erlotinib (**C**) and U87 EGFRvIII cells with dacomitinib (**D**). * denotes *p*-value was less than 0.05 when S1 and S3 values were compared, & denotes *p*-value was less than 0.05 when values of S2 and S3 were compared, and # denotes when *p*-value was less than 0.05 when S1 and S2 values were compared.

EGFR inhibition studies were designed after analyzing the results obtained from the FAK phosphorylation inhibition studies. We observed that the stiffness sensitive cells shared the unique feature of either having EGFR upregulation or an EGFR mutation. We hypothesized that these cells, which sense their environment through FAK phosphorylation, leading to increased proliferation rates on hydrogels of increased ***E’***, may also be sensitive to intervention in the EGFR pathway. We tested this hypothesis by inhibiting EGFR and assessing whether this would replicate the results that observed in the FAK phosphorylation inhibition studies. The drugs employed in these studies were the EGFR inhibitors erlotinib (Selleck chem) for U373 cells and U87 cells, and dacomitinib (Selleck chem) for A172 cells and U87 EGFRvIII cells. Erlotinib is known to be active against wild-type EGFR seen in U87 cells and EGFR upregulation seen in U373 cells [51], while the EGFR mutations in A172 cells and U87 EGFRvIII cells have been shown to be resistant to erlotinib [52]. Hence dacomitinib, which has been shown to target the mutations seen in these cell lines, was chosen [53]. The concentrations used were ten times their IC 50 concentrations, which were 20 nM for erlotinib and 60 nM for dacomitinib. The protocol for the defactinib study was repeated, with these drugs in place of defactinib. Media was supplemented with the appropriate drug for the entire seven days of the experiment, and media and drug replenishment was performed on day four. In Figure 4, proliferation on the 3 ***E’*** values under EGFR inhibition using erlotinib in U373 cells and U87 cells, and dacomitinib for U87EGFRvIII and A172 cells, is demonstrated. The aberrant EGFR expressing cells exhibit a similar reversal, as seen in the proliferation assays with the FAK phosphorylation inhibitor, defactinib. U87 cells lose their stiffness sensitivity, which was the same result obtained when FAK phosphorylation was blocked.

Our hypothesis that the cells deduce the tumor microenvironment ***E’*** through the FAK phosphorylation appears to be correct from the results presented in Figure 2 and Figure 3. The ***E’*** preference of the cells could be manipulated and reversed in certain cases when FAK phosphorylation was blocked*.* Moreover, our immunohistochemical studies also show that FAK phosphorylation increases in cells as hydrogel ***E’*** of increased. It is important to note that the all of the cell lines that displayed this level of sensitivity and experienced reversals in their ***E’*** preference had either upregulation of EGFR, as in U373 cells, or EGFR mutations, as in A172 and U87 EFGRvIII [21,52]. Previous studies conducted in this area indicate there is a co-clustering of EGFR and focal adhesion complexes when U373 is cultured on a stiff environment [40]. In the case of U-87 cells, it was shown that the downstream of EGFR signaling was activated in the stiffer environment [53]. There are various experiments in the past lending credit to the important connection between mechanosensing, FAK, and EGFR signaling [54]. It was demonstrated that FAK inhibition in glioblastoma was shown to sensitize the tumor to EGFR inhibition [54]. In the case of U87 EGFRVIII cells, it has also been shown that FAK phosphorylation and the EGFRvIII pathway are connected, and blocking FAK phosphorylation has been shown to decrease proliferation [50]. Our experiments showed that the EGFR inhibition of the cells having a predilection for higher ***E’*** hydrogels caused the cells to reverse stiffness preference, and proliferate faster on lower ***E’*** hydrogels. Exhibition of similar behavior during FAK phosphorylation strongly suggests that the EGFR and FAK pathways are interlinked in these cell populations, as suspected. Likely, the FAK and EGFR pathways are interconnected in a way such that when one pathway is activated, it can lead to the activation of the other pathway [22]. Table 1 summarizes of the results obtained from all the elastic moduli-based proliferation assays.

### 2.4. Dynamic Elastic Modulus Reduction as a Therapeutic Application

**Figure 5:**
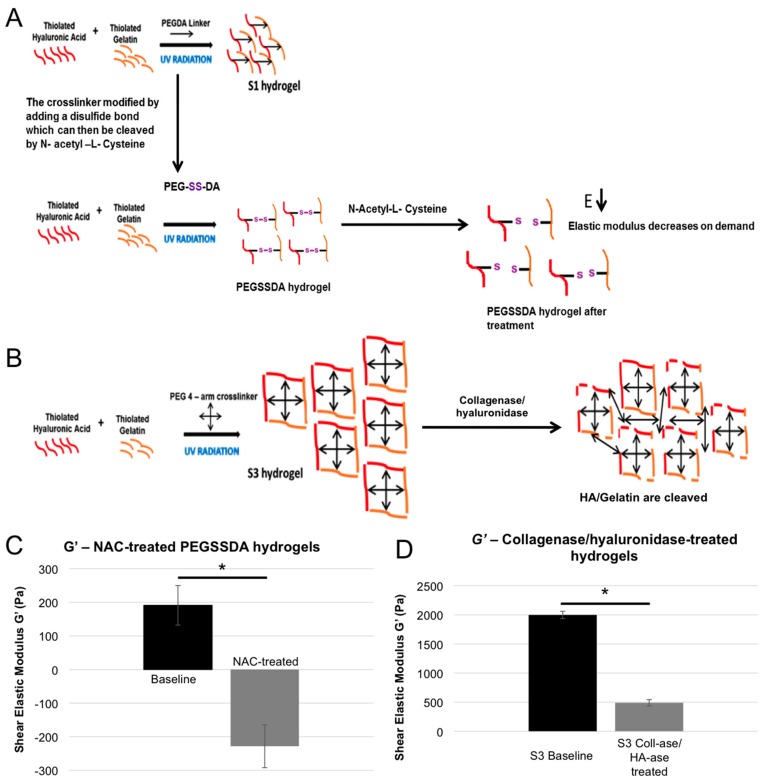
(**A**) The integration of a double sulfide bond in the cross-linker, which can then be broken on demand and thereby decrease the elastic modulus of the hydrogels on demand; (**B**) The elastic modulus reduction experiment modified to suit in vivo conditions, in which hydrogels without the breakable cross-linker are partially digested using collagenase/hyaluronidase to decrease the elastic modulus; (**C**,**D**) Rheological data demonstrating NAC treatment reduction PEGSSDA cross-linked hydrogels and collagenase/hyaluronidase treatment reduction of S3 hydrogels, respectively. Statistical significance: * *p* < 0.01.

The above experiments demonstrated that the proliferation of GBM populations of particular genotypes show substantial sensitivity to the elastic modulus of their environment. Thus, we hypothesized that this dependence on the physical environment could be harnessed for diagnostic and therapeutic purposes. Specifically, could targeting the elastic modulus of the tumor microenvironment and dynamically reducing it lead to a reduction in tumor cell proliferation? To test this hypothesis, we employed a variation of the hydrogel that is cross-linked with a breakable PEG cross-linker, thereby providing a hydrogel system with crosslinks that could be reduced dynamically at a later point in time [39]. In this system, the cross-linker is broken through a reduction process where a disulfide bond in the cross-linker can be cleaved by the addition of *N*-acetyl-l-cysteine (NAC) (Figure 5A,C). This cleavage allows the elastic modulus of the gel to be decreased on demand by the addition of the compound on day 4 of the proliferation study time course, after which the relative proliferation rates could be compared to hydrogels in which the ***E’*** had not been dynamically reduced.

**Figure 6:**
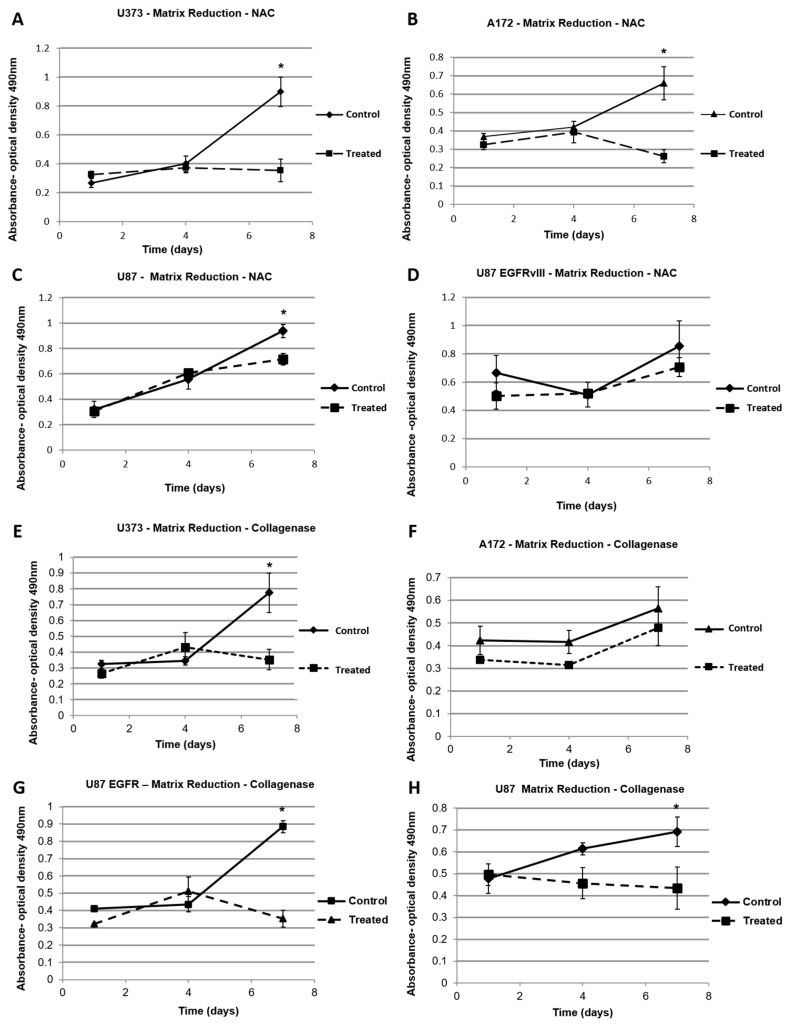
Targeting elastic modulus by using *N*-Acetyl-l-Cysteine to break disulfide bonds in PEGSSDA cross-linked hydrogels, and resulting proliferation rates: (**A**) U373 cells; (**B**) A172 cells; (**C**) U87 cells and (**D**) U87 EGFRvIII cells. Targeting elastic modulus with collagenase/hyaluronidase to degrade the hyaluronic acid (HA) and gelatin components in S3 hydrogels: (**E**) U373 cells; (**F**) A172 cells; (**G**) U87 cells and (**H**) U87 EGFR viii cells. * Denotes *p* value is less than 0.05.

We conducted proliferation assays with hydrogels designed to fail using a breakable disulfide bridge containing PEGSSDA cross-linker. The elastic modulus of the hydrogels was decreased on demand with *N*-acetyl-l-cysteine when needed. In Figure 6A–D, it can be observed that the proliferation rates of U373, A172, U87 and cells have decreased once the elastic modulus of the PEGSSDA hydrogels was decreased using NAC. However, there was no notable reduction in the proliferation rate of U87 EGFRvIII cells when the elastic modulus was reduced.

As the brain has no breakable cross-linker, we decided to create an approach that would replicate the in vivo condition, thus simulating how a therapeutic ECM degradation event might be achieved. Therefore, we used the stiffer S3 hydrogels, and their elastic moduli were decreased on demand with collagenase/hyaluronidase (Figure 5B,D). In Figure 6E–H, it can be observed that the proliferation rates of U373, A172, U87 and U87 EGFRvIII cells have decreased once the elastic modulus of the S3 hydrogels was decreased using collagenase/hyaluronidase.

**Figure 7:**
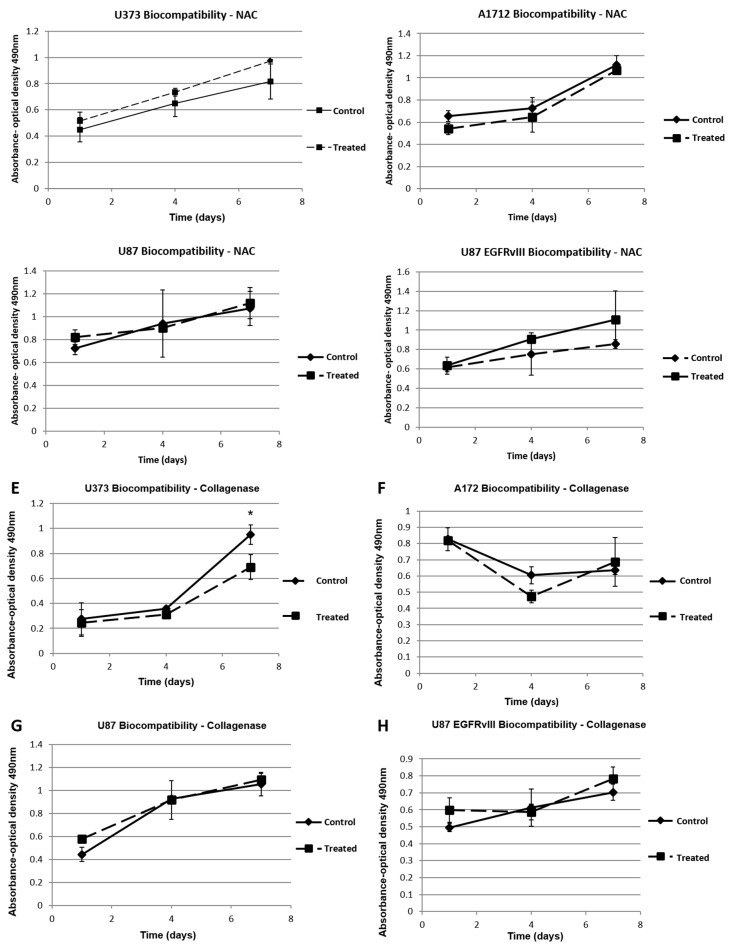
Biocompatibility assays of cells with *N*-Acetyl-l-cysteine (**A**) U373 cells; (**B**) A172 cells; (**C**) U87 cells and (**D**) U87 EGFRvIII cells. Biocompatibility assays of cells with collagenase/hyaluronidase; (**E**) U373 cells; (**F**) A172 cells; (**G**) U87 cells and (**H**) U87 EGFRvIII cells. Other than the case of U373 cells under collagenase treatment (* *p* < 0.05), control and treated conditions are not significantly different, indicating that the NAC and collagenase/hyaluronidase treatments do not contribute to a reduction in cell proliferation in the ***E’*** reduction studies.

Following the NAC and collagenase/hyaluronidase-based ***E’*** reduction studies, we wanted to confirm that the proliferation rates of the cells that decreased were due to the effect of reduced ***E’*** of the hydrogels on the cells, and not any toxic effects of the NAC or collagenase/hyaluronidase. We conducted biocompatibility tests with the cells cultured on S3 hydrogels without the breakable cross-linker, and treated the cells with NAC on day 4. We also conducted collagenase/hyaluronidase biocompatibility tests with cells cultured on tissue culture plastic, as the enzyme would reduce any of the gel conditions. Again, the cells were treated on day 4. The results of these assays show that the NAC and collagenase/hyaluronidase treatments do not cause significant changes in cell proliferation compared to non-treated control conditions (Figure 7).

Our elastic modulus manipulation experiments indicate that the proliferation and invasiveness of the tumor cells could be curtailed with the reduction of the elastic modulus of the hydrogels. We demonstrated these effects using two methodologies. First, we used a cross-linker engineered to contain a disulfide bridge, which serves as a site that can be chemically targeted and broken by NAC. This dynamic covalent chemistry allows formation of the hydrogel constructs, and subsequent elastic modulus reduction at a later time, after cells have been in culture for some time. Second, we utilized collagenase/hyaluronidase to directly degrade the HA and gelatin components of the hydrogel. In both scenarios, for most of the cell populations tested, this reduction strategy succeeded in slowing or completely stopping tumor cell proliferation. As the reagents used for reducing the hydrogel elastic moduli are biocompatible, the decrease in tumor cell proliferation is solely due to the dynamic decrease of the elastic modulus of the hydrogels on which these cells were cultured. In future work, we aim to explore this concept further with a preclinical/clinical focus, as these results suggest that tumor ECM elastic modulus reduction could be potentially used as a therapeutic target where the native HA of the brain could be targeted, reducing the tumor microenvironment ***E’***, and subsequently slowing or preventing tumor growth.

Prior research to ascertain the effect that the elastic modulus of the physical microenvironment has on GBM cells has been mostly done with substrates such as polyacrylamide, collagen, and Matrigel—materials that do not mimic brain ECM. Only a few recent studies have used HA-based hydrogels [40,55,56,57]. These studies compared the proliferation rates and motility of cells at different elastic moduli, and demonstrated that most GBM cells proliferated at a higher rate with increased motility on stiffer substrates [40,55,56], but only among one or two GBM cell lines. It has been shown that elevated FAK expression has been implicated to play a major role in the promotion of GBM cell proliferation [17]. We have taken this one step further by showing gradual FAK phosphorylation increases on hydrogels with different elastic moduli, and have shown that cells have higher rates of FAK phosphorylation on stiffer hydrogels, thus corroborating the earlier findings mentioned above. Additionally, EGFR has been demonstrated to be a key player in the increasing proliferation rates of the cells on stiffer substrates [40]. A direct link between FAK and the EGFR signaling pathway has been established by showing that inhibiting FAK phosphorylation could sensitize the cells to EGFR inhibition in several cancer type cell lines, including glioblastoma [54,58]. We have expanded on these findings to study the effect of FAK phosphorylation inhibition and EGFR inhibition in cell lines with EGFR mutation or EGFR upregulation when cultured on stiffer substrates. The stiffness sensitivity of GBM cell lines with unique EGFR mutations, as in A172 (190 kDa EGFR) and U87 EGFRviii (170 kDa), on hyaluronic-based hydrogels had not been explored prior to this study [21]. Notably, we also tested the novel concept of targeting the stiffness sensitivity of GBM cells as a translatable therapeutic option by conducting dynamic matrix reduction experiments, facilitated by the dynamic chemical reactions supported by our HA hydrogel system.

## 3. Conclusions

The results of these studies suggest that there is a strong correlation between EGFR mutation and FAK phosphorylation in EGFR-aberrant GBM populations. The mechanisms of this cross-talk and activation are not completely understood and will require additional studies. For example, EGFR stimulation of GBM cells with FAK knockout or, conversely, FAK stimulation in EGFR knockouts could be a next step in understanding the complex interconnections between FAK signaling and the EGFR pathway. Additionally, we would like to apply the methodologies described in these studies to patient GBM biopsy-derived cell populations. Importantly, the results described here, as well as other studies [33], indicate that some GBM populations prefer substrates of higher elastic modulus, indicating that they are stiffness-sensitive, while others do not. So, while tumor microenvironment elastic modulus sensitivity may not be relevant to every patient with GBM, the interplay between physical parameters and genetic profiles may serve as a type of actionable biomarker. This is particularly important as new diagnostic tools emerge. For example, with the advances in magnetic resonance elastography (MRE), the link between mutation identification and elastic modulus parameters could be a relatively straightforward diagnostic scenario that could lead to novel and effective therapies. MRE is a rapidly developing technology for quantitatively assessing the mechanical properties of tissue [59]. Recent studies using MRE indicate that the elastic modulus of different brain tumors can be different, and the elastic modulus of normal brain tissue is different from the cancerous tumor tissue [60]. This technology could potentially be used in combination with genetic analysis to diagnose and determine optimal therapies for particular patients with brain tumors such as GBM. In the feasibility studies described here using a tunable dynamic hydrogel system, we show that GBM subpopulations respond to environmental ***E’*** changes differently. Cells with aberrant EGFR expression accelerate their proliferation in stiffer environments. The inhibition of either EGFR or focal adhesion kinase, or performing a dynamic reduction in tumor microenvironment matrix stiffness, significantly slowed tumor growth of the EGFR mutants. Based on these results, we believe that novel therapies can be developed in the future that do not necessarily target the cells in the tumor, but rather target the tumor microenvironment extracellular matrix to slow or prevent the progression of GBM tumors.

## 4. Materials and Methods

### 4.1. Cells Employed in Elastic Modulus Experiments

Glioblastoma cell lines U-373 MG (ATCC^®^ HTB-16™) U-87 MG (ATCC^®^ HTB-14™) (obtained from Cell and Viral Vector Laboratory Shared Resource of Wake Forest Baptist Medical Center, Winston-Salem, NC, USA), U-87EGFRvIII cell line (gifted by Webster Cavenee from Ludwig Cancer Research Institute, San Diego, CA, USA) and A172 (ATCC^®^ CRL-1620™) (obtained from ATCC, Manassas, VA, USA) were employed for all experiments. All the cancer cell lines were cultured in Dulbecco’s Modified Eagle Medium (DMEM) high glucose with 10% FBS, 4 mM l-glutamine and 200 U/mL penicillin and 200 ug/mL streptomycin in a tissue culture incubator at 37 °C with 5% CO_2_.

### 4.2. Hydrogels

HyStem-HP hydrogels (ESI-BIO, Alameda, CA, USA) were employed to create the tunable tumor microenvironment in our system. These hydrogels were chosen because the primary component is HA. The components of the hydrogel include thiol-modified HA with heparin pendant chains (Heprasil), thiol-modified gelatin (Gelin-S) and a thiol-reactive PEGDA cross-linker (Extralink-molecular weight of 3400 kDa). Heprasil was reconstituted with distilled water to produce a 1% weight by volume solution and both Gelin-S, and Extralink was reconstituted with 0.5% photoinitiator (Irgacure D-2959 photoinitiator, Sigma Aldrich, St. Louis, MO, USA) to produce 1% weight by volume solution. The reconstituted vials were placed in a 37 °C incubator for 40 min for the solids to completely dissolve and form a clear solution. A second cross-linker, PEG 4-ARM acrylate with a molecular weight of 10,000 kDa (Creative PEGWorks, Winston-Salem, NC, USA), was used to manipulate the elastic modulus of the hydrogels.

Three different elastic modulus realms were created for this experiment using the hydrogels and cross-linker combinations. The ***E’*** values of the hydrogels that were created were 100, 1000 and 2000 Pa. These formulations were created by manipulating the cross-linkers. To create the S1 hydrogels, Heprasil, Gelin-S, and Extralink were mixed 2:2:1 by volume. To create the S2 hydrogels, Heprasil, Gelin-S, Extralink, and PEG 4–ARM acrylate were mixed 2:2:0.5:0.5 by volume. To create the S3 hydrogels, Heprasil, Gelin-S, and PEG 4–ARM acrylate were mixed 2:2:1 by volume. For use in experiments, the mixed hydrogel components were instantaneously photopolymerized using UV irradiation at 365 nM for 1–2 s. ***E’*** values were validated by rheological procedures from previous studies using a Discovery Hybrid DH2 Rheometer (TA Instruments, New Castle, DE, USA) as described [36,38,45].

### 4.3. Proliferation Assays

In a 96-well plate, 10,000 cells of each cell type were placed on 50 μL of UV cross-linked hydrogels. All experimental conditions were tested in triplicate or higher. The cells were cultured on the hydrogels with DMEM-10 media for one week in an incubator (5% CO_2_, 37 °C). On day 1, 4 and 7, we conducted MTS assays (CellTiter 96^®^ Aqueous One Solution Cell Proliferation Assay (MTS), Promega, Madison, WI, USA), providing absorbance values based on mitochondrial metabolism proportional to cell number. For these assays, media was removed from three wells of each condition, and 200 μL of MTS media (170 μL of media mixed with 30 μL of MTS reagent) was placed and incubated for one hour. Then 100 μL of the media was removed from these wells and then placed in a clean 96-well plate. Absorbance was quantified with a SpectrumMax M5 optical plate reader (Molecular Devices, Sunnyvale, CA, USA) at 490 nm.

### 4.4. Immunofluorescence Staining for FAK Phosphorylation

Immunofluorescence staining was performed in chamber slides rather than well plates. Ten thousand cells of each cell line queried (U373, A172, and U87 EGFRvIII) were placed on the cross-linked hydrogels in the chamber slides. The S1, S2 and S3 hydrogels were used to determine whether there was a difference in the expression of FAK phosphorylation of the cells based on hydrogel ***E’***. The chamber slides were filled with DMEM-10 media and maintained in culture for three days before the cells were fixed. The cells were fixed with 4% paraformaldehyde for 1 h, and permeabilized with phosphate-buffered saline with Tween 20 for 1 h. The cells were then blocked with protein block from Abcam for 4 h. The primary antibody phospho-FAK (Tyr397) ABfinity Rabbit monoclonal (1 in 400 dilution, raised in rabbit, ThermoFisher, Waltham, MA, USA) was applied at room temperature for 4 h. Next, a secondary anti-rabbit Alexa Fluor 594-conjugated antibody (1 in 400 dilution, ThermoFisher, Waitham, MA, USA) was used in U373 and A172 wells, while a secondary anti-rabbet Alexa Fluor 488-conjugated antibody (1 in 400 dilution, ThermoFisher) was used in U87 EGFRvIII wells. Secondary antibody incubation was performed for 4 h at room temperature. Finally, DAPI (1 in 500 dilution) was applied to all the cells for 15 min, and the cells were imaged in a Zeiss Axiovert 200M (Carl Zeiss AG, Oberkochen, Germany) at 100× magnification.

### 4.5. FAK and EGFR Inhibition

In a 96-well plate, 10,000 cells of each cell type were placed on 50 μL of UV cross-linked hydrogels. For inhibition of FAK phosphorylation, cells were cultured with DMEM-10 media with Defactinib (VS-6063, PF-04554878) for a week in an incubator (5% CO_2_, 37 °C). All experimental conditions were tested in triplicate or higher, and MTS assays were performed as described above on day 1, 4 and 7. Defactinib was added to DMEM media at a concentration of 100 nM, and the media was replenished in all the wells on day 4. For inhibition of EGFR, cells were cultured with DMEM-10 media with the appropriate EGFR inhibitor drug. The drugs used for EFGR inhibition were erlotinib (Selleckchem, Houston, TX, USA) for U373 cells and U87 cells, and dacomitinib (Selleckchem) for A172 cells and U87EGFRvIII cells. The concentrations used were ten times their IC 50 concentrations, which were 20 nM for erlotinib and 60 nM for dacomitinib. A media change was performed on day 4 to replenish the drugs in the media.

### 4.7. Hydrogel Reduction Through a Breakable Cross-linker

S3 hydrogels were prepared in a 96-well plate: nine wells for control conditions and nine wells that were treated with NAC for each cell line. The hydrogels were cross-linked with PEGSSDA (Polyethylene glycol disulfide diacrylate), as mentioned above. The 20 μL of Heprasil (mixed with Distilled H20), 20 μL of Gelin-S (mixed with photoinitiator) were mixed with 10 μL of PEGSSDA cross-linker (mixed with photoinitiator) and then cross-linked with UV light. Ten thousand cells of each cell line were placed in each of the wells. The wells were filled with media, and then the plates were placed in the incubator and cultured for a week. MTS assays were performed on day 1, 4 and 7. On day 4, the three wells that would be assayed on day 7 were treated with 50 mM of NAC solution for 2 h. The wells were then rinsed well, and then were again filled with media. The control wells were also rinsed with media in parallel. MTS assays were then performed for both of the conditions as described above.

### 4.8. Hydrogel Reduction through Enzymatic Degradation

S3 hydrogels were prepared as described above, without the breakable cross-linker. The NAC-experiment above was essentially replicated, except a collagenase/hyaluronidase enzyme (StemCell Technologies, Vancouver, CA, USA) was employed to decrease the elastic modulus of the hydrogels by partially degrading the hyaluronan polymer chains and gelatin proteins in the hydrogels. The same experimental design described for the NAC experiments was used. Collagenase/hyaluronidase was diluted by a factor of 10 in serum protein-free media. On day 4, the media from three wells were removed and rinsed well with serum-free media three times, exposed to 200 μL of 10× diluted collagenase in serum-free media for 5 min, and then quenched with normal DMEM media with serum proteins to stop the action of the enzyme. The control wells were also given media changes in parallel. MTS assays were again performed on day 1, 4 and 7; the results were analyzed as described for previous assays.

### 4.9. NAC and Collagenase/Hyaluronidase Biocompatibility Verification

Proliferation assays were prepared with the four cell types on 50 μL of S3 hydrogels cross-linked with non-breakable PEG cross-linkers in a 96-well plate. Ten thousand cells were seeded in each well in triplicate for each day and each condition (control and NAC treatment), and MTS assays were performed on day 1, 4 and 7. On day 4, the treatment group was treated with 200 μL of 50 mM NAC for 1 h, followed by a media change. The control group wells were also given media changes in parallel.

Collagenase biocompatibility was assessed similarly, except cells were seeded directly in a 96-well plate. The hydrogel could not be used for this validation study, as it would be degraded by the collagenase. MTS assays were performed on day 1, 4 and 7. On day 4, the treatment group was treated with 200 μL of collagenase 10× diluted with serum-free DMEM for 5 min, followed by serum-containing DMEM. The control group wells were also given media changes in parallel.

### 4.10. Statistical Analysis

All quantitative results are presented as mean ± standard deviation (SD). Experiments were performed in triplicate or greater. Values were compared using Student’s *t* test (2-tailed) with two sample equal variance, and *p* < 0.05 or less was considered statistically significant.

## Figures and Tables

**Table 1:** Summary of results of elastic modulus versus proliferation assays under no drugs, defactinib, and EGFR inhibition. Statistical significance: * *p* < 0.05 when S1 and S3 values were compared; ^&^ *p* < 0.05 when values of S2 and S3 were compared.

Stiffness Sensitivity Summary			
Cell Line	Proliferation Rate Highest in Normal Condition	Proliferation Rate Highest in Defactinib	Proliferation Rate Highest in EGFR Inhibition
**U373-MG**	S3 *^,&^	S1 *	No distinct preference
**A172**	S2 ^&^ and S3 * ^(DAY 4)^	S1 *	S1 * and S2 ^&^
**U87-MG**	S2 ^&^	S2	No distinct preference
**U87-EGFRvIII**	S3	S1	S1

## Supplementary Materials

The following are available online at www.mdpi.com/2310-2861/3/3/28/s1, Figure S1. Comparisons for (A) U373, (B) A172, (C) U87, and (D) U87 EGFRvIII cell proliferation on the 3 hydrogel formulations (S1, S2, and S3) and on tissue culture plastic (96-well plate). To account for anticipated increased proliferation rates on plastic, 3 cell densities were employed in the plastic cultures. In each cell population, in general, proliferation was significantly increased on plastic (* *p* < 0.05 between all conditions at day 7 in hydrogel cultures versus plastic cultures). The exceptions to this statistical difference are the 10,000 cell cultures on plastic using A172, U87, and U87 EGFRvIII cells. In these wells, cells reached confluence quickly and thus the growth curve plateaued.
