# Supplementary material for: Exploration of Dynamic Elastic Modulus Changes on Glioblastoma Cell Populations with Aberrant EGFR Expression as a Potential Therapeutic Intervention Using a Tunable Hyaluronic Acid Hydrogel Platform
Source: Gels. 2017 Jul 13;3(3):28. doi: 10.3390/gels3030028 (PMC6318698; doi:10.3390/gels3030028)
Supplement: Supplementary file 1 [file gels-03-00028-s001.pdf]

# Exploration of Dynamic Elastic Modulus Changes on Glioblastoma Cell Populations with Aberrant EGFR Expression as a Potential Therapeutic Intervention Using a Tunable Hyaluronic Acid Hydrogel Platform

**Hemamylammal Sivakumar, Roy Strowd and Aleksander Skardal**

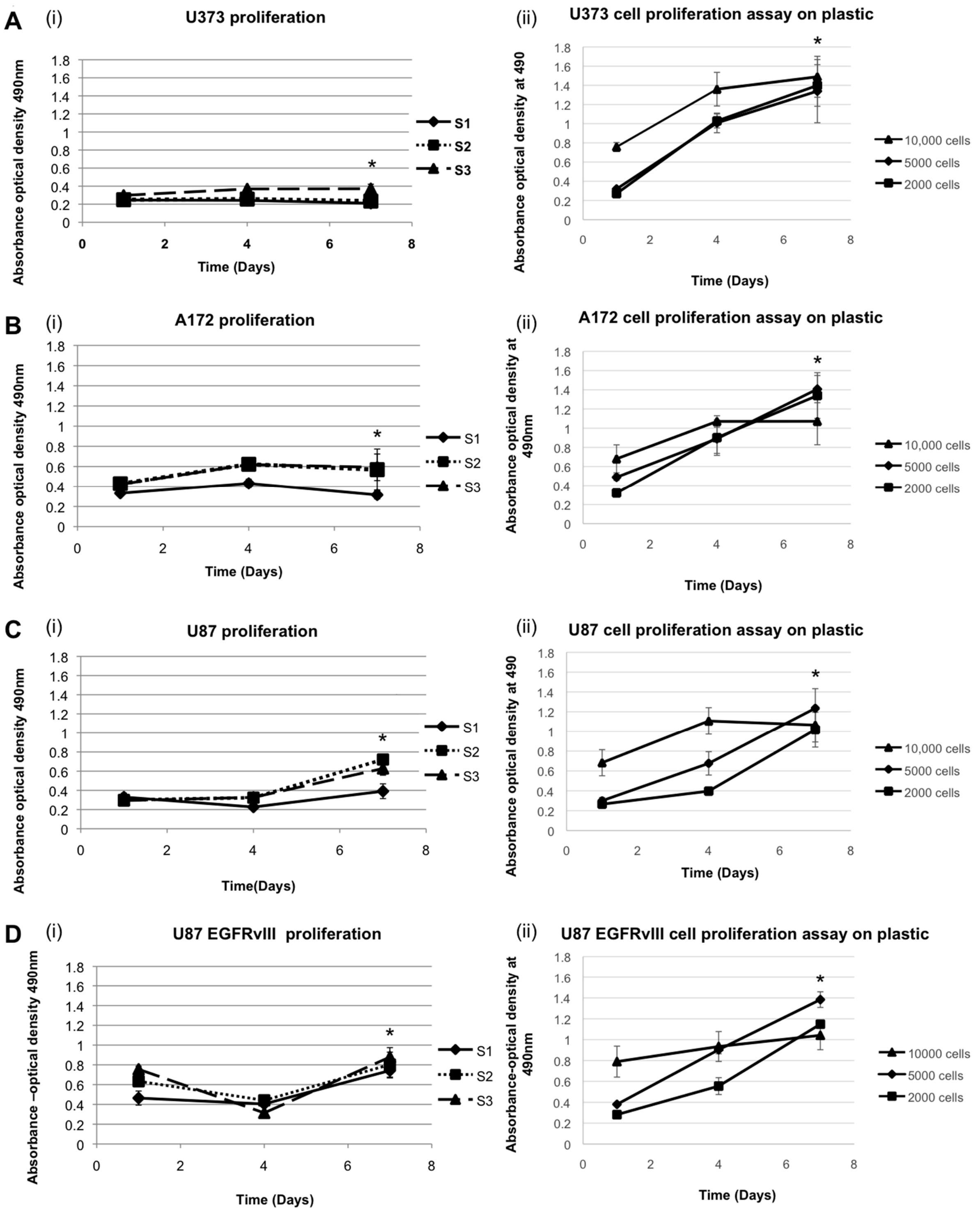


**Figure S1.** Comparisons for (**A**) U373, (**B**) A172, (**C**) U87, and (**D**) U87 EGFRvIII cell proliferation on the 3 hydrogel formulations (S1, S2, and S3) and on tissue culture plastic (96-well plate). To account for anticipated increased proliferation rates on plastic, 3 cell densities were employed in the plastic cultures. In each cell population, in general, proliferation was significantly increased on plastic (* $p < 0.05$ between all conditions at day 7 in hydrogel cultures versus plastic cultures). The exceptions to this statistical

difference are the 10,000 cell cultures on plastic using A172, U87, and U87 EGFRvIII cells. In these wells, cells reached confluence quickly and thus the growth curve plateaued.
